# Oxidant Stress, Hyperoxia/Hypoxia and Neonatal Respiratory Disorders

**DOI:** 10.3390/antiox14121389

**Published:** 2025-11-21

**Authors:** Ourania Kaltsogianni, Theodore Dassios, Anne Greenough

**Affiliations:** 1 Women and Children’s Health, School of Life Course and Population Sciences, Faculty of Life Sciences and Medicine, King’s College London, London SE5 9RJ, UK; ourania.kaltsogianni@nhs.net (O.K.); theodore.dassios@kcl.ac.uk (T.D.); 2 Neonatal Intensive Care Centre, King’s College Hospital NHS Foundation Trust, London SE5 9RS, UK

**Keywords:** oxidative stress, neonate, hyperoxia, hypoxia

## Abstract

Neonates, especially those born prematurely, have low antioxidant capacity and are highly exposed to oxidant stress during the perinatal period. Oxidant stress damage has been associated with several diseases of prematurity, including respiratory distress syndrome (RDS), bronchopulmonary dysplasia (BPD), and pulmonary hypertension. In addition, preterm infants are frequently exposed to hypoxia or hyperoxia, which further increases oxidant stress and morbidity. This narrative review describes the relationship between oxidant stress, hyperoxia/hypoxia, and neonatal respiratory disorders. Preterm infants with respiratory distress syndrome and BPD have higher levels of oxidative stress biomarkers in plasma and in tracheal aspirates and reduced activity of antioxidant enzymes. Early, prolonged, and frequent intermittent hypoxaemic episodes are related to BPD development. Exposure to hyperoxia is linked to longer duration of respiratory support and higher BPD rates. Preclinical data showed that intermittent hypoxia and hyperoxia are associated with pulmonary hypertension (PH) and that hyperoxia can negatively affect the response to pulmonary vasodilators. Antioxidant treatments are not routinely implemented into clinical care due to their modest effect on clinical outcomes, associated complications, and limited clinical data. Optimisation of oxygen delivery and monitoring with closed-loop automated oxygen control systems could potentially reduce oxidant stress in the neonatal environment.

## 1. Introduction

Reactive oxygen species (ROS) are a group of molecules that are formed from molecular oxygen through reduction–oxidation or electronic excitation. These molecules can be classified as free radicals and non-radicals. ROS are generated endogenously or as a result of environmental exposure, and several mechanisms exist to control their production and availability. Under physiological conditions, ROS function as second messengers that regulate cellular processes through different mechanisms, including changes to enzyme activity, gene transcription, and membrane integrity [1]. The imbalance between increased ROS production and deficient intracellular antioxidant systems leads to the generation of free radicals and subsequent cell damage or disrupted redox signalling, a condition frequently described as oxidant stress [2]. More recently, the term “oxidative distress” has been used to describe this imbalance to account for the presence of oxidant stress and for the importance of redox signalling under physiological conditions [3]. A key metabolite in oxidant stress is hydrogen peroxide (H_2_O_2_), which is derived from molecular oxygen by NADPH oxidases, superoxide dismutases (SOD), and the mitochondrial electron transport chain in mammalian cells with aerobic metabolism [3]. Biological signalling is mainly mediated by oxidation of sulfur in target proteins and affects several cellular responses, including cell proliferation, migration, differentiation, and angiogenesis [4]. Antioxidant enzymes (catalases, glutathione peroxidases, and peroxiredoxins) are responsible for the removal of H_2_O_2_ [5]. Higher levels of H_2_O_2_ lead to nonspecific protein oxidation and altered signalling, which cause damage to all cellular components and subsequently lead to inflammation, growth arrest, and cell death [1].

Free radicals are reactive oxygen, nitrogen, or sulfur species that contain one or more unpaired electrons [6]. Free radicals are a product of metabolic redox reactions, mainly in the respiratory chain, as a consequence of exogenous and endogenous processes such as hypoxia, ischemia, ischemia–reperfusion, inflammation, hyperoxia, neutrophil and macrophage activation, and mitochondrial dysfunction [7]. Free radicals are unstable molecules that react with cellular components and affect cellular and tissue functions, leading to organ damage. Antioxidants are produced endogenously or potentially can be administered exogenously and protect from free radicals by either neutralising or removing them and by repairing the free-radical-induced cellular damage (Table 1) [8,9].

Pregnancy complications and conditions, including pre-eclampsia, diabetes, undernutrition, smoking, infection or inflammation, may trigger an acute increase in free radical formation and, thus, create an adverse intrauterine environment that affects foetal development [10]. Foetal oxidant stress has also been demonstrated in pregnancies complicated by intrauterine growth restriction (IUGR), as this condition is often linked with impaired placental blood flow and intrauterine hypoxia that causes the generation of free radicals [11]. It has also been hypothesised that foetal oxidant stress may be the underlying mechanism that links placental dysfunction to foetal programming that predisposes to adult diseases such as metabolic syndrome, obesity, diabetes, hyperinsulinemia, hypertension, and cardiovascular disease [10]. An impaired oxidant/ antioxidant status, as demonstrated by significantly higher isoprostane and prostaglandin F2 alpha levels, was found in children born small or large for gestation when compared with those who had an appropriate for gestation birth weight [12]. In addition, animal studies link foetal oxidant stress to renal and cardiovascular alterations, which contribute to hypertension, endothelial dysfunction, ventricular hypertrophy, and susceptibility to develop arrhythmias [13].

Newborn infants are susceptible to significant oxidant stress during the transition from the hypoxic intrauterine environment to the relatively high oxygen exposure after birth. This transition, in combination with the use of supplementary oxygen during resuscitation, assisted ventilation, administration of surfactant, total parenteral nutrition, and blood transfusions, further enhances the production of free radicals and exacerbates oxidant stress [5].

Newborn infants have decreased antioxidant capacity and are highly exposed to oxidant stress. Antioxidant defence mechanisms progressively increase during gestation, and many important vitamins and minerals with antioxidant roles are delivered to the foetus during the third trimester of pregnancy [14]. Premature birth interrupts the synthesis and passage of maternal antioxidants and, consequently, increases the susceptibility of preterm infants to oxidant stress damage, which has been associated with several diseases of prematurity [14].

Neonatal patients, and especially those born prematurely, experience frequent fluctuations of their transcutaneous oxygen saturation (SpO_2_) levels, either due to respiratory pauses or apnoea and ineffective breathing efforts in spontaneously breathing infants [15]. Other physiologic parameters that contribute to desaturations are the low functional residual capacity (FRC) of preterm infants, which results from atelectasis and high chest wall compliance, and low blood oxygen capacity, including blood volume and haemoglobin content [16]. In ventilated infants, episodes of intermittent hypoxia (IH) occur due to ineffective respiratory support and loss of lung volume [17]. In a cohort study, extremely preterm infants experienced up to 600 intermittent hypoxic episodes within one week, and these were associated with a higher risk of developing retinopathy of prematurity (ROP) requiring laser treatment [18]. In addition, newborn infants receiving supplemental oxygen are frequently exposed to hyperoxia and its associated complications. Studies on the achievement of oxygen saturation targets in extremely preterm and very low birth weight (VLBW) infants showed that they spent 20% to 73% of the time in hyperoxia [19,20].

**Figure 1:**
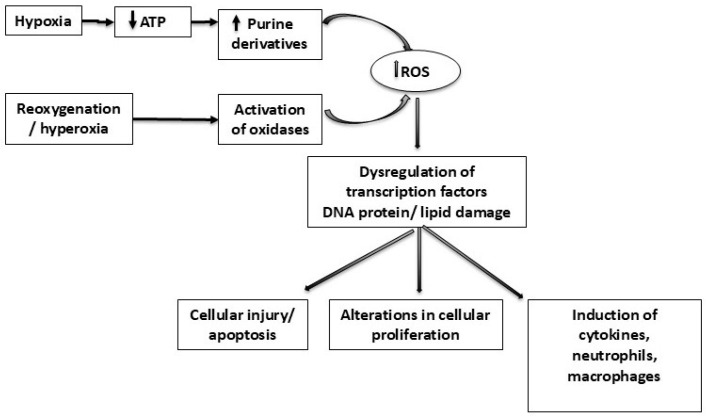
Diagram demonstrating the generation of reactive oxygen species (ROS) relative to hypoxia and hyperoxia in neonatal respiratory disorders. Hypoxia leads to adenosine triphosphate (ATP) exhaustion and subsequent increased formation of purine derivatives. Reoxygenation and excess oxygen/hyperoxia activate oxidases that metabolise purine derivatives and generate reactive oxygen species (ROS) (e.g., superoxide anion) that cause oxidative stress and tissue damage. ATP: adenosine triphosphate; DNA: deoxyribonucleic acid; ROS: reactive oxygen species.

Hypoxia, hyperoxia, and fluctuations between these conditions increase oxidant stress and generate reactive oxygen species that result in alterations in cellular proliferation and apoptosis (Figure 1) [21]. Hypoxaemic events during early postnatal life have been associated with multiple poor outcomes, including growth restriction [22], sleep-disordered breathing [23], ROP [24], neurodevelopmental impairment [24], and mortality [24]. Hyperoxia has been related to lung, retina, central nervous system (CNS), and red blood cell injuries, as well as generalised tissue damage in the neonatal period [25]. Recurrent IH and hyperoxia exposure may result in long-term changes in respiratory mechanics with increased airway resistance and decreased compliance and may contribute to wheezing in former preterm infants [26].

Early exposure to oxidative stress has been associated with altered lung development processes and susceptibility to neonatal respiratory disorders, including respiratory distress syndrome (RDS), bronchopulmonary dysplasia (BPD), and persistent pulmonary hypertension (PH) [27]. This narrative review describes the relationship between oxidant stress, hyperoxia/ hypoxia, and neonatal respiratory disorders.

## 2. Respiratory Distress Syndrome

Respiratory distress syndrome (RDS) is the most common respiratory disorder in preterm infants. Among other factors, oxidative stress plays a significant role in the pathophysiology of RDS. Preterm infants at birth frequently experience periods of hypoxia and reoxygenation that lead to an increase in oxidant stress. ROS increase proinflammatory cytokines, activate neutrophils, and lead to an acute inflammatory state with subsequent alveolar injury and pulmonary damage [28]. Bodha et al. found lower levels of reduced glutathione (GSH) in the tracheal aspirates of preterm infants with RDS compared with controls that were electively intubated for surgery. In addition, oxidised glutathione (GSSG) and the ratio of GSSG to GSH were significantly increased and correlated with disease severity [29]. Dizdar et al. showed an association of lower serum total antioxidant capacity (TAC) levels with longer duration of respiratory support in preterm infants with RDS. Surfactant administration significantly increased TAC levels and improved oxidant–antioxidant balance [30]. A pilot study demonstrated a significant increase in the plasma levels of interleukin (IL)-6, IL-8, and tumour necrosis factor (TNF)-α in preterm ventilated infants with high oxygen requirements (FiO_2_ > 0.3) compared with infants requiring lower inspired oxygen concentration (FiO_2_ < 0.3) [31]. In addition, in a subsequent case–control study, preterm infants with RDS (n = 31) had significantly higher plasma levels of oxidative stress markers (malondialdehyde (MDA) and hydrogen peroxide (H_2_O_2_)) and reduced activity of antioxidant enzymes (catalase (CAT) and superoxide dismutase (SOD)) when compared with healthy preterm newborns (n = 36) [32]. However, more infants in the RDS group were born by caesarean section, which may also increase oxidative stress [33].

## 3. Bronchopulmonary Dysplasia

Despite advances in neonatal care, bronchopulmonary dysplasia (BPD) is one of the most common serious morbidities of prematurity and is associated with adverse respiratory and neurodevelopmental outcomes [34]. BPD is a multifactorial disease, and its pathogenesis entails an ongoing process of injury and recovery of an immature lung [35]. Intermittent hypoxaemic episodes have the potential to trigger a proinflammatory cascade [36] and oxidative stress [37], which have both been implicated in the pathogenesis of BPD. Chronic oxygen toxicity damages the pulmonary epithelium, inactivates surfactant, and causes intra-alveolar oedema and interstitial wall thickening with fibrosis, leading to pulmonary atelectasis. This results in disturbed alveolarisation, which is a pathological characteristic of BPD [25]. Inflammatory markers, including neutrophils, interleukin 8 (IL-8), and elastase, were increased in the bronchoalveolar lavage fluid of infants with BPD, supporting the role of inflammation and oxidative stress in the development of the disease [38]. In addition, IL-6, as a marker of airway inflammation, and 8-hydroxy-20-deoxyguanosine (8-OHdG), which indicates oxidative DNA damage, were higher in tracheal aspirates of preterm ventilated infants with BPD on postnatal days 1 and 28 compared with preterm infants without BPD [39]. Among 104 preterm infants born at less than 30 weeks of gestation, higher carboxyhaemoglobin (COHb) levels from blood gas analysis in the first seven days of life were significantly associated with the risk of developing BPD [40]. The level of COHb is a marker of endogenous production of carbon monoxide as a consequence of oxidative stress [41].

Fairchild et al. studied episodes of bradycardia or hypoxaemia (SpO_2_ < 80% for ≥10 s) in 502 VLBW infants, 37% of whom developed BPD, in the first four weeks after birth. The frequency and duration of hypoxaemic episodes overall and during the fourth week were significantly associated with the development of BPD [42]. In a retrospective cohort of 137 extremely preterm infants, a diagnosis of BPD at 36 weeks postmenstrual age was associated with increased daily frequency of IH episodes, longer duration, and a higher IH nadir from day seven to day 26 of life. Increased numbers of IH events occurring 1–20 min apart were also related to this outcome. Infants with BPD had a lower mean SpO_2_ and higher mean airway pressure and inspired oxygen concentration requirements [43]. Similarly, a post hoc analysis of the Canadian Oxygen Trial that included 1018 extremely preterm infants demonstrated significant differences in hypoxaemic exposure among infants who developed BPD compared to those who did not, and these differences increased in magnitude over the first four weeks of life. Both the frequency of prolonged intermittent hypoxic episodes (SpO_2_ < 80% and duration of ≥60 s) and the proportion of time per day with SpO_2_ < 80% between birth and 36 weeks postmenstrual age (PMA) were significantly associated with the risk of severe BPD [44]. In the same cohort of extremely preterm infants, intermittent hypoxic episodes were linked to increased risk of late death or childhood disability at 18 months corrected age [24]. BPD increases the risk of adverse neurodevelopment [45]. Therefore, early and prolonged intermittent hypoxic episodes may independently contribute to an increased risk of poor neurodevelopmental outcomes and BPD.

Oxygen toxicity has been well recognised as one of the factors contributing to the development of BPD. Animal models suggest that even brief exposure to hyperoxia can cause long-term morphologic and functional changes in the lung [46,47], resulting in a phenotype comparable with BPD [47]. Those data are in agreement with clinical studies showing that restricted use of supplemental oxygen or lower SpO_2_ targets is associated with a reduction in lung inflammation and BPD rates [48]. In a prospective observational study in 502 VLBW infants, the implementation of lower SpO_2_ targets (85 to 93% versus 92 to 100%) reduced the incidence of BPD and the use of postnatal corticosteroids; the number needed to treat was six [49].

Vento et al. randomised 78 extremely preterm infants to a lower (0.3) or higher (0.9) fraction of the inspired oxygen concentration during resuscitation at birth. Patients in the lower FiO_2_ group required significantly fewer days of supplemental oxygen, mechanical ventilation, and continuous positive airway pressure support and had lower BPD rates (15.4% versus 31.7%). In addition, infants who received higher FiO_2_ were characterised by increased expression of proinflammatory cytokines and oxidative stress biomarkers in the first week after birth, and those indices correlated significantly with BPD development [50]. Birenbaum et al. showed a 50% reduction in the incidence of BPD among 145 VLBW infants with the use of lower oxygen concentrations during resuscitation and lower oxygen saturation targets (88–92%). However, the study was limited by its retrospective design and several changes implemented into the neonatal care during the study period that prevented the authors from being able to accurately attribute the relative influence of each factor on the outcome of interest [51].

## 4. Pulmonary Hypertension

During the intrauterine period, the foetus is subjected to a relative hypoxaemia, which results in a physiological state of pulmonary hypertension (PH). At birth, lung aeration leads to a rise in oxygen tension that contributes to rapid pulmonary vasodilation. Failure of the pulmonary vasculature to relax results in increased pulmonary arterial pressure and vascular resistance with right-to-left shunting of deoxygenated blood from the pulmonary to the systemic circulation and the development of persistent pulmonary hypertension of the newborn (PPHN) [52]. Oxygen is often used in the treatment of PPHN as a potent pulmonary vasodilator. Preclinical studies have shown that even short-lasting alveolar hyperoxia can be harmful for newborns with PPHN, as it seems to increase pulmonary vascular contractility [53] and impairs the subsequent response to pulmonary vasodilators, including inhaled nitric oxide (iNO) [54]. In a lamb model of PPHN, a gradual increase in the FiO_2_ up to 0.5 significantly reduced the pulmonary vascular resistance, but no additional decrease was observed with a further increase in the oxygen concentration to 100% [54].

The alveolar and lung vascular changes in infants with BPD may also result in an increase in pulmonary vascular resistance and subsequently in the development of PH [55]. Animal data suggest that intermittent hypoxia is implicated in the pathogenesis of PH. In a rat model of BPD, IH during recovery from hyperoxia-induced lung injury prevented recovery of alveologenesis and increased pulmonary vascular resistance, the Fulton index (which is the weight ratio of the right ventricle to the sum of the left ventricle and septum [56]), and arterial wall thickness [57]. Exposure of neonatal mice to IH from day seven of postnatal life and for three weeks resulted in altered lung endothelial cell function, mitochondrial DNA lesions, and impaired lung angiogenesis. When superimposed on hyperoxia, IH induced a severe lung vascular phenotype that is seen in preterm infants with PH [58]. In a case–control study that included 80 extremely preterm infants with BPD, prolonged severe hypoxaemic episodes (SpO_2_ < 70% and <80%) were associated with PH and increased mortality [59].

## 5. Antioxidant Treatments

Many antioxidant therapeutic strategies have been studied to determine if they would reduce oxidative stress in newborn infants. Vitamins A and E have been extensively investigated as potential antioxidant therapies for the prevention of BPD with regard to whether they could eliminate lipid peroxidation induced by ROS. A Cochrane review and meta-analysis included 10 randomised controlled trials (RCTs) and 1460 infants comparing vitamin A with a control and showed a marginal reduction (risk reduction (RR): 0.87, 95% confidence interval (CI): 0.77 to 0.99) in the incidence of BPD at 36 weeks PMA and no significant differences in neurodevelopmental outcomes at 18–22 months corrected age [60]. Vitamin A supplementation has not been implemented into standard clinical practice due to its modest effect and the need for intramuscular administration [61]. A more recent systematic review and meta-analysis showed that enteral compared with parenteral vitamin A is equally effective in reducing BPD rates at 36 weeks postmenstrual age, but the benefit was limited to infants with low baseline intake (<1500 IUIU/kg/day) [61]. Vitamin E was not found to play a significant role in the prevention of BPD (RR = 0.659, 95% CI = 0.243–1.786; *p* = 0.412) [62]. Vitamin E supplementation may have a neuroprotective effect [63] and reduce severe ROP, but high intravenous doses in VLBW infants have been associated with increased risk of sepsis [64].

Vitamin C (ascorbic acid: AA) is a water-soluble antioxidant in cells and plasma that is actively transported across the placenta [65]. In addition, in vitro studies demonstrate a prooxidant activity via the Fenton reaction [66]. Evidence regarding the association between AA concentration and respiratory morbidity in preterm infants is conflicting. In a prospective observational study that included 144 infants of any gestation, higher AA concentrations on day two of life were associated with a higher risk of developing BPD [67]. A double-blind RCT that included 119 VLBW infants did not show any significant association between AA levels throughout the first 28 days of life and the incidence of BPD at 36 weeks postmenstrual age. Higher AA supplementation was associated with a trend towards reduced need for supplemental oxygen at 36 weeks postmenstrual age [68]. A more recent systematic review and meta-analysis included two studies (160 VLBW infants) and demonstrated a significant reduction in the risk for BPD with vitamin C supplementation but with very low certainty evidence [69]. In another systematic review and meta-analysis, vitamin C supplementation in pregnancies exposed to smoking was found to reduce the incidence of wheeze in offspring at 12 months and five years. No significant association was found between vitamin C and E supplementation and the risk for RDS [70].

Superoxide dismutase (SOD), an antioxidant enzyme, converts the superoxide radical that is generated through metabolism or other cell reactions to hydrogen peroxide (H_2_O_2_) and molecular oxygen (O_2_). Antioxidant enzymes, including catalase, glutathione peroxidase, and peroxiredoxins, break down H_2_O_2_ to water and molecular oxygen [5]. SOD is decreased in the pulmonary vascular endothelium of preterm infants with BPD, leading to reduced vasodilation induced by nitric oxide [71]. Studies in lamb models of PPHN demonstrated that intratracheal administration of recombinant human SOD (r-h SOD) reduced oxidant stress [72,73] and improved nitric oxide-mediated vasodilation [72]. In an RCT in preterm infants with RDS, intratracheal administration of r-h SOD every 48 h during mechanical ventilation (MV) for up to one month of age did not significantly affect the rates of death or BPD at 36 weeks postmenstrual age. At follow-up, however, infants treated with r-h SOD had fewer wheezing episodes and respiratory illnesses requiring hospitalisation compared with controls when assessed at one year of age [74].

Breast milk consists of many antioxidants, including vitamins, SOD, glutathion, melatonin, and short-chain fatty acids [75]. Therefore, in addition to its other advantages with regard to nutrition and immunity, it may contribute to reducing oxidative stress for newborn infants. Human milk following preterm birth has been found to have higher antioxidant capacity when compared to term milk and may help reduce the incidence of oxidative stress-related diseases of prematurity [76]. A systematic review and meta-analysis included 15 studies (n = 4984 infants) evaluating the effect of mother’s own milk on the incidence of BPD. They found that breast milk as an exclusive diet was associated with a significant reduction in the risk of BPD (RR: 0.74, 95% CI: 0.57–0.96, five studies) when compared with preterm formula, regardless of gestation at birth [77]. A recent review concluded that the antioxidant capacity of breast milk may be the primary mechanism through which it contributes to reduced BPD rates [75]. These results further emphasise the well-established benefits of breast milk for preterm infants.

Novel antioxidant therapies aim at the induction of endogenous antioxidant systems, as, following systemic administration, they may not reach the target cells where oxidative stress causes injury [78]. One example is melatonin, a neurohormone that induces several endogenous antioxidant systems. In an RCT that included 110 infants < 32 weeks of gestation, intravenous administration of melatonin in divided doses and within six hours of life led to significant reductions in inflammatory cytokines and ventilator and oxygen requirements compared with controls. Furthermore, none of the infants treated with melatonin were oxygen dependent at 28 days of life, whereas there were six infants (7.5%) with oxygen dependency in the control group [79]. These promising results need to be examined in a larger RCT, preferably with long-term follow-up.

A recent review highlighted the limitations of therapeutic interventions in targeting oxidative stress [80]. Oxidative stress is usually a contributor to the disease rather than its primary cause, and this limits the effectiveness of antioxidant treatments. In addition, agents that appear to work in vitro may not provide the same results in vivo due to difficulty in achieving adequate concentrations [80]. Therapeutic strategies should selectively target disease-related mechanisms to avoid disrupting some important signalling processes mediated by oxidants [1]. Preventing oxidant production or inhibiting oxidant-induced signalling and increasing the synthesis of endogenous antioxidant enzymes should be the focus of emerging antioxidant therapies [80].

## 6. Closed-Loop Automated Oxygen Control Systems

Closed-loop automated feedback-controlled (CLAC) systems of oxygen delivery monitor oxygen saturation values in real time to calculate and make an adjustment to the inspired oxygen concentration, without any human intervention [81]. Several algorithms have been developed to provide automated oxygen control in preterm infants. All of them use SpO_2_ levels measured by pulse oximetry as an input, but they process this information in different ways in order to adjust the inspired oxygen concentration. CLAC has been extensively studied in very preterm or low birth weight infants. The available studies demonstrated that CLAC monitoring increases the time spent within the target SpO_2_ range, improves the stability of partial arterial oxygen pressure (PaO_2_), and reduces the time spent in hypoxia and hyperoxia, with fewer manual adjustments to the inspired oxygen concentration [81,82,83,84,85,86]. Results appear to be consistent for all the control algorithms [86,87], across different SpO_2_ target ranges [87], and in infants receiving either mechanical ventilation or non-invasive respiratory support [85]. In addition, CLAC facilitates earlier weaning of the FiO_2_ compared with manual oxygen control [88,89]. In a randomised crossover study in ventilated preterm infants, hourly median FiO_2_ values were lower during the automated period (median 24 h FiO_2_: 0.32 versus 0.37, *p* < 0.001) and infants spent longer time with FiO_2_ < 0.25 (median % time with FiO_2_ < 0.25: 8% versus 0%, *p* = 0.018) [89]. Reynolds et al. [88] demonstrated that preterm infants receiving high-flow nasal cannula oxygen spent more time in air during automated oxygen control when compared with manual control [88]. However, there is limited evidence on the effect of CLAC on longer-term respiratory and other outcomes [81,85,86]. The majority of studies had a randomised crossover design and were not sufficiently powered to detect significant differences in long-term outcomes. A recent Cochrane review [85] included 18 randomised trials, which compared CLAC with manual oxygen control or different systems of automated oxygen control in preterm infants and concluded that none of the studies assessed time on oxygen therapy, BPD, severe ROP, necrotising enterocolitis, or mortality [85].

The improvement in the achievement of oxygen saturation targets and the reduction in the duration of supplemental oxygen treatment could ameliorate the risks related to hypoxia and hyperoxia and improve neonatal respiratory morbidity. We have recently reported that, in an RCT in preterm ventilated infants, CLAC monitoring was associated with significant reductions in the duration of MV and supplemental oxygen treatment, incidences of BPD and moderate or severe BPD or death, and the need for home oxygen [90]. These promising results should be further explored in large multicentre studies before the routine implementation of CLAC in clinical practice.

## 7. Conclusions

Newborn infants, especially those born prematurely, are exposed to oxidative stress, which increases their susceptibility to respiratory disorders and other free-radical-related diseases of prematurity. Oxygen is the most used drug in neonatal intensive care, and inappropriate dosing may lead to hypoxia or hyperoxia that further increases oxidative damage. Strategies to limit oxidant stress need to be developed.

Despite promising early data, no specific antioxidant treatments are currently used in the neonatal setting. Major barriers to effective treatments include the lack of targets of the antioxidant factors, their limited bioavailability to the tissue in which oxidative stress is ongoing, and the lack of knowledge regarding appropriate dosing, as well as the timing of therapy, as there is evidence of oxidative stress occurring even in the perinatal period. Further, the genetic variability of the subjects may affect their susceptibility to oxidative stress and response to treatment [91]. Genetic differences between antioxidant systems may influence individual responses to hypoxia and hyperoxia. In a cohort of 284 VLBW infants with BPD, genetic variants of a transcription factor that induces antioxidant enzymes (nuclear factor erythroid-2 related factor-2 (NFE2L2)) were significantly associated with the risk and severity of BPD after adjusting for epidemiological confounders [92]. Therefore, genomic sequencing may allow for the identification of individual differences in endogenous antioxidant systems and the development of tailored therapeutic interventions.

The complex interplay between oxidant stress and antioxidants has recently been reviewed [1,80]. These reviews highlight that the results of clinical trials in this field are rather disappointing and emphasise that a greater understanding of the mechanisms of action of antioxidants is necessary.

Future studies should explore the biomarkers that would help identify patients at high risk of oxidative injury at an early stage and develop novel, tailored therapies.

Importantly, the implementation of strategies that will promote an optimal perinatal environment with minimal risk of oxidative stress appears necessary. As oxygen can cause oxidative damage, the optimisation of oxygen delivery and monitoring should also be the focus of future research. CLAC systems appear promising in reducing the risks related to oxygen treatment. Future research should further explore the effects of CLAC on clinical outcomes and evaluate its implementation in clinical practice.

## Figures and Tables

**Table 1:** Major oxidants and antioxidants.

**Oxidants**	
*Reactive oxygen species*	
	Superoxide (O_2˙_^─^)
	Hydrogen peroxide (H_2_O_2_)
	Hydroxyl radical (HO˙)
	Hypochlorite (HOCl)
*Reactive nitrogen species*	
	Nitric oxide (NO)
	Nitric dioxide (NO_2˙_)
	Nitric trioxide (NO_3_˙)
	Nitrate (NO_3_^─^)
	Peroxynitrite (ONOO˙)
*Reactive sulfur species*	
Radicals	Thiyl radical (RS˙)
	Glutathionyl radical (GSSG˙)
	Reactive sulfane species (RSR˙)
Non-radicals	Reactive sulfane species (RSR)
	Reducing sulfur species (hydrogen sulfide (H_2_S), reduced glutathione (GSH))
	Reactive sulfur substances (sulfur dioxide (SO_2_), sulfur trioxide (SO_3_))
	Sulfur secondary metabolites (Allicin)
**Antioxidants**	
*Enzymatic*	Primary enzymes: superoxide dismutase, catalase, glutathione peroxidase
	Secondary enzymes: glutathione reductase, glucose-6-phosphate-dehydrogenase
*Non- enzymatic*	Flavonoids
	Cofactors: coenzyme Q10
	Minerals: zinc, selenium
	Organosulfur compounds: glutathione
	Vitamins and derivatives: A (retinol), C (Ascorbic acid), E (tocopherol), K
	Carotenoids: β-carotene, lycopene, lutein, zeaxanthin
	Nitrogen non-protein compounds: uric acid
	Phenolic acids: hydroxycinnamic acids, hydroxybenzoic acids

## Data Availability

No new data were created or analyzed in this study. Data sharing is not applicable to this article.

## Abbreviations

The following abbreviations are used in this manuscript:

AA	Ascorbic acid
BPD	Bronchopulmonary dysplasia
CAT	Catalase
CLAC	Closed-loop automated oxygen control
CNS	Central nervous system
COHb	Carboxyhaemoglobin
FiO_2_	Fraction of inspired oxygen
FRC	Functional residual capacity
GSH	Reduced glutathione
GSSG	Oxidised glutathione
H_2_O_2_	Hydrogen peroxide
IH	Intermittent hypoxia
IL	Interleukin
iNO	Inhaled nitric oxide
IUGR	Intrauterine growth restriction
MDA	Malondialdehyde
MV	Mechanical ventilation
NFE2L2	Nuclear factor erythroid-2 related factor-2
8-OHdG	8-hydroxy-20-deoxyguanosine
PH	Pulmonary hypertension
PMA	Postmenstrual age
PaO_2_	Partial arterial oxygen pressure
PPHN	Persistent pulmonary hypertension of the newborn
RCT	Randomised controlled trial
RDS	Respiratory distress syndrome
ROP	Retinopathy of prematurity
ROS	Reactive oxygen species
SOD	Superoxide dismutase
SpO_2_	Peripheral oxygen saturation
TAC	Total antioxidant capacity
TNF	Tumour necrosis factor
VLBW	Very low birth weight
